# Inflammation-mediated facial nodules to compounded tirzepatide

**DOI:** 10.1016/j.jdcr.2026.06.026

**Published:** 2026-06-17

**Authors:** Luke C. Moore, Kara Hurley, Angela Yen Moore

**Affiliations:** a Arlington Center for Dermatology, Arlington, Texas; b Arlington Research Center, Arlington, Texas; c McGovern Medical School at UTHealth Houston, Houston, Texas; d Department of Dermatology, Texas Tech University Health Sciences Center, Lubbock, Texas; e Texas Christian University Anne Burnett Marion School of Medicine, Fort Worth, Texas; f Baylor University Medical Center, Dallas, Texas

**Keywords:** adverse event, facial filler, nodule, tirzepatide

*To the Editor:* We appreciate Pitak-Arnnop’s interest in proposing an infectious etiology for the facial nodules after tirzepatide as opposed to an inflammatory 1.^1^ The letter reiterated many of the limitations raised in the paper, especially the lack of histological and microbiological confirmation. While a biopsy would be ideal to rule out an infectious etiology, it was not performed due to the nodules' location on the face in cosmetically sensitive areas and the patient’s refusal.

Nonetheless, we believe that inflammatory etiology is most likely. One of the most significant factors in establishing the most likely etiology is the lack of response to doxycycline. Pitak-Arnnop’s letter implies improvement with doxycycline, which indeed would support an infectious etiology. However, the patient did not improve with 2 rounds of doxycycline, which suggests an alternative etiology. Improvement with isotretinoin supports an inflammatory etiology rather than an infectious 1.^2^

Finally, Pitak-Arnnop’s letter postulates that injections at different clinical sites by different practitioners would increase infection risk. Multiple injectors across different medical clinics are unlikely to introduce similar infectious contaminants into filler injection site—distant from the compounded tirzepatide injection site. Additionally, no new facial nodules occurred after ceasing compounded tirzepatide even after subsequent filler injections. A subsequent rechallenge with compounded tirzepatide a few weeks after filler injections resulted in delayed onset of new nodules at previous injection sites; this temporal association implicates compounded tirzepatide from an inflammatory pathway rather than an infectious pathway.

### Declaration of generative AI and AI-assisted technologies in the writing process

No generative AI was used in the manuscript composition.

## Conflicts of interest

None disclosed.
